# Associations between ubiquitin, follicle-stimulating hormone, and sex steroid hormones in the failed to conceive female dromedary camels raised in hot climates

**DOI:** 10.14202/vetworld.2022.2046-2051

**Published:** 2022-08-24

**Authors:** Yousef Mesfer Alharbi

**Affiliations:** Department of Veterinary Medicine, College of Agriculture and Veterinary Medicine, Qassim University, Buraydah, Saudi Arabia

**Keywords:** camel, estrogens, follicle-stimulating hormone, progesterone, subfertility, ubiquitin

## Abstract

**Background and Aim:**

The reproductive management of female dromedary camels involves traditional implications that are widespread among desert camel raisers. Several subfertility clinical manifestations impede pregnancy and elongate the interval between parturitions. Ubiquitin is a novel-specific protein, referred to recently as a biomarker for reproductive performance in male and female mammals. Therefore, this study aimed to investigate the association between subfertility clinical status and the peripheral levels of ubiquitin versus follicle-stimulating hormone (FSH), progesterone, and estradiol.

**Materials and Methods:**

According to the clinical diagnoses, 80 female dromedaries admitted to the university clinic were categorized into six female groups suffering from endometritis (EN, 28; 35%), inactive ovaries (IO, 18; 22.5%), ovarian hydrobursitis (BU, 19; 23.75%), vaginal adhesions (VA, 7; 8.75%), salpingitis (SA, 4; 5%), and cervicitis (CE, 4; 5%). In addition, five normal fertile non-pregnant females served as controls (CONs). All animals underwent ultrasonography and blood sampling for hormone and ubiquitin determinations.

**Results:**

The results revealed a significant (p < 0.05) increase in ubiquitin in the CE (577.22 pg/mL) and VA (670.92 pg/mL) females. However, lower ubiquitin levels but still higher than the CON were noted in females with other symptoms (225.76, 425.79, 394.02, 414.96, and 393.92 pg/mL in the CON, BU, SA, IO, and EN, respectively). Concomitantly, the mean levels of FSH revealed a similar trend, showing higher (p < 0.05) levels in CE (2.79 mIU/mL) and VA (2.5 pg/mL) females. In contrast, no change was observed in FSH among other groups than CON (2.11, 2.17, 2.01, 2.24, and 2.13 mIU/mL in CON, BU, SA, IO, and EN, respectively). There was no difference in the progesterone levels among groups; however, estradiol-17ß levels significantly differed (p < 0.01), showing the highest level (629.15 pg/mL) in the SA group with no significant difference among other groups.

**Conclusion:**

Thus, ubiquitin could be used as a biomarker for genital tract inflammation in female camels raised in hot climates.

## Introduction

It is well known that heat stress affects an animal’s productivity and welfare. Camels are considered elemental and serve as backbones for a large number of Bedouins living in the Arabian Peninsula. Camels not only contribute as human feed due to its highly nutritious meat and milk but also form part of a rich heritage in the Arabian Gulf region. Raising camels in drought-stricken and arid regions impose burdens on the animal’s overall productivity. Moreover, the reproductive management of such animals is plagued by several traditional malpractices that illiterate caretakers employ. These induced several injuries to the female genital tracts [1, 2].

The most common reproductive concerns among female camels include ovarian-bursal pathology [3], endometritis (EN), salpingitis (SA), vaginal infections and inflammation, cervicitis (CE), and ovarian inactivity [4]. Hurley (2015) [5] elucidated that bacterial colonization of the lower reproductive tract in cows post-parturition promotes the action of neutrophils and macrophages to direct epithelial regeneration of the uterine lining; however, this could elicit an increase in cytokines in the blood. Although ubiquitin has been identified and characterized since 1975 as a peptide of 96 amino acids (8.5 kDa), its definite role in reproduction has not yet been elucidated until the 21^st^ century. Earlier, ubiquitin was referred to as a peptide involved in male and female gametogenesis, modulation of steroid receptor concentrations, placental development, and endometrial preparation for fetal implantation [6]. Recently, however, ubiquitin was found to be involved in cellular inflammation [7]. Inflammation is defined as physiological changes in the body tissues as a response to injurious factors, that are, injuries, bruises, burns, infections, scratches, etc. As the pathogen invades the body, the immune system is triggered. It recruits many inflammatory cells that secrete various types of cytokines and inflammatory mediators, resulting in inflammation [8]. The inflammatory response could result in tissue adhesions and tumors and promote metastasis [9]. Several studies have been conducted on the beneficial roles of ubiquitin on the reproductive performance of males [10] and females [11]. However, limited evidence has been attributed to ubiquitin and its function in reproductive failure in domestic animals.

Therefore, this study aimed to elucidate the relationship between follicle-stimulating hormone (FSH), progesterone, and estradiol-17ß as the leading players in female reproduction and ubiquitin concentrations in the peripheral blood of infertile female dromedaries raised in hot and drought climates. Moreover, we will attempt to evaluate the causes of infertility in female camels via ubiquitin monitoring as a biomarker of inflammation.

## Materials and Methods

### Ethical approval

The study was approved by Committee for ethical handling, welfare, and use of animals, Qassim University under contract # 7804-Q1-1-2019.

### Study period and location

The study was conducted from February to July 2020 at Qassim University Educational Veterinary Hospital.

### Animals and location

**Figure-1:**
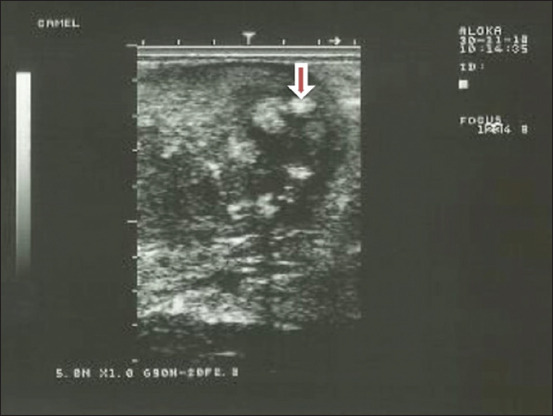
Uterine inflammation (endometritis) in dromedary camel female (Purulent materials intrauterine – arrow).

**Figure-2:**
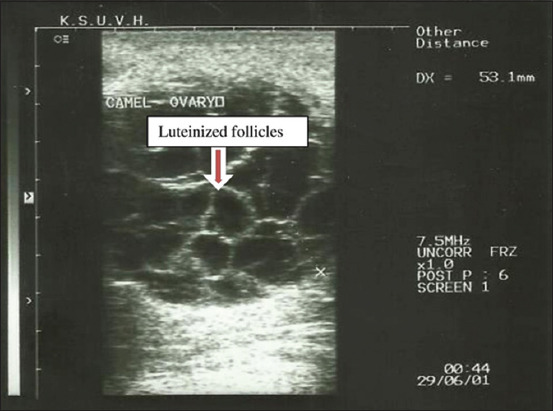
Ovarian cysts (luteinized follicles) in dromedary camel females.

**Figure-3:**
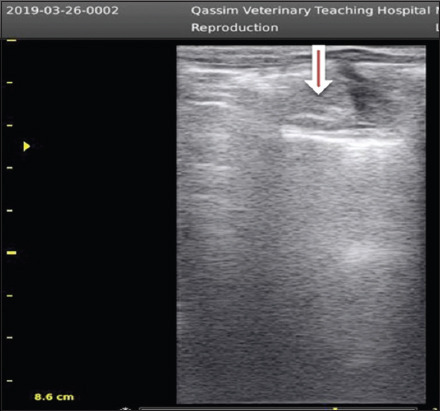
Smooth (inactive) ovary in dromedary camel female (arrow).

**Figure-4:**
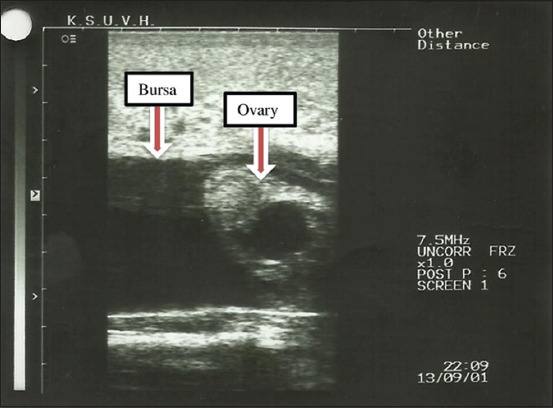
Ovarian hydrobursa in dromedary camel female.

**Figure-5:**
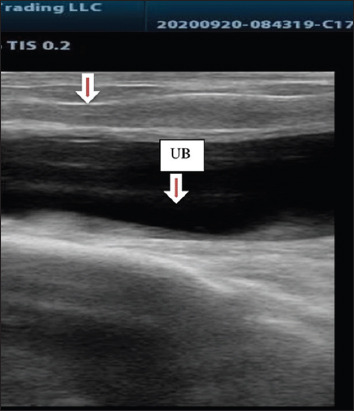
Purulent material in the vagina (vaginitis) in dromedary camel female (arrow), (UB = urinary bladder).

**Figure-6:**
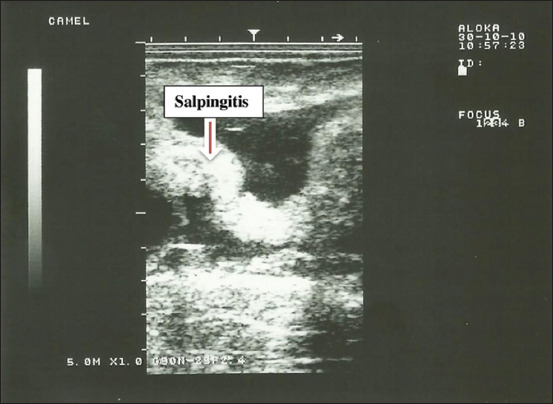
Salpingitis (fallopian tube adhesion) in dromedary camel female.

**Figure-7:**
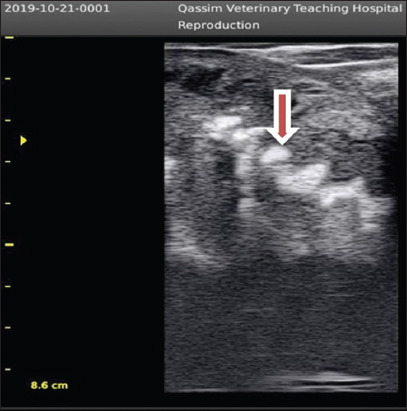
Purulent material intracervical (cervicitis) in dromedary camel female (arrow).

Eighty-two female dromedary camels were admitted to the veterinary educational clinic for various reproductive complaints. The reproductive tract of each animal was examined using standard transrectal palpation and by ultrasonography attached to a 5 MHz probe (Aloka SSD 500; Aloka Co., Ltd., Tokyo, Japan). The vagina was examined manually with a gloved hand to estimate the patency of the vagina and cervix and to evaluate the nature of the vaginal discharges. All animals (including control [CON]) were bled by jugular veni­puncture (10 mL) into non-heparinized Vacutainer^®^ tubes. EN (Figure-1), cystic ovaries (Figure-2), inactive ovaries (IO) (Figure-3), ovarian hydrobursitis (BU) (Figure-4), vaginitis (Figure-5), salpingitis (Figure-6), and CE (Figure-7) were diagnosed in 28 (34.14%), 20 (24.39%), 19 (23.17%), 7 (0.53%), 4 (4.87%), and 4 (4.87%) of the barren females, respectively. Furthermore, five normally cycling non-pregnant females were served as CONs and subjected to blood sampling.

### Serum harvesting

Blood samples were transported to the laboratory, left for 2 h in the refrigerator, and then centrifuged at 800× *g* for 10 min; sera were harvested in clean labeled tubes and kept frozen (−20°C) until assayed for the designed parameters.

### Ubiquitin, FSH, estradiol-17ß, and progesterone determination

#### Camel ubiquitin determination

Camel ubiquitin cross-reactive protein was determined using a commercial sandwich enzyme-linked immunoassay (ELISA) kit (My BioSource, San Diego, CA, USA). The intra-assay C.V is 9.2%. The lower detection limit was found to be at 62.5/mL, and the sensitivity was 10 pg/mL. The standard curve was linear with R^2^ = 0.98.

#### Camel FSH determination

Camel FSH was determined using a commercial sandwich ELISA kit specific for camels (My BioSource). The assay sensitivity was 0.1 mIU/mL. The detection range of the assay was 0.625–20 mIU/mL; however, the intra-assay CV was 10.7%.

#### Estradiol-17ß determination

A commercial competitive ELISA kit (Human Gesellschaft für Biochemica und Diagnostica mbH, Germany) was used to determine estradiol concentration in camel serum. The assay sensitivity was 13 pg/mL, the detection range was 25–2000 pg/mL, and the intra-assay CV was 8.4%.

#### Progesterone determination

Progesterone concentrations in camel sera were determined using a commercial competitive ELISA kit (Human Gesellschaft für Biochemica und Diagnostica mbH). The sensitivity of the assay was 0.03–0.07 ng/mL, the detection range was 0.3–40 ng/mL, and intra- and inter-assay CV were 6.5% and 9.7%, respectively.

### Statistical analysis

Hormone and ubiquitin data were analyzed using a one-way analysis of variance of the general linear model by Statistical Analysis System (SAS) software version 9.0 PROC GLM of SAS [12].

The mean comparisons among groups were performed using Duncan’s multiple range test, and statistical significance was set at p < 0.05. Pearson’s correlations among hormones were estimated within groups [12]. The statistical model is described as follows:

Y_ij_ = μ + A_i_ + e_ij_

Where; Y_ij_ is the observation taken on the j^th^ individual,

μ = overall mean,

A_i_ = the fixed effect of the i^th^ fertility status, (I = 1….7).

e_ij_ = random error assumed to be independent normally distributed with mean = 0 and variance = Ợ^2^.

## Results

The veterinary educational clinic receives several camels suffering from various infertility issues. Most of the concerns raised by the camel owners center around repeated breeding but without conception. A total of 82 infertile female camels were categorized into six groups according to the clinical diagnosis depending on the cause of infertility. Results reveal a significant (p < 0.05) reduction in the peripheral blood levels of FSH in all infertile groups than in the fertile CON (3.38 mIU/mL). The second two groups showing high levels of FSH were females with cervical and vaginal inflammation. The ubiquitin concentration reveals to be lowest (p < 0.05) among fertile females (205.04 ng/mL), while it is highest among females with vaginal (670.92 ng/mL) and cervical (577.22 ng/mL) inflammation. All other infertile groups showed ubiquitin levels higher than the CON (Table-1).

**Table-1:** Association between the subfertility status of the female dromedary camel and blood ubiquitin, FSH, estradiol-17ß, and progesterone (Mean ± SEM).

Female group	No. of female	FSH (mIU)	Hormone/peptide		

			Ubiquitin (pg/mL)	Estradiol (pg/mL)	Progesterone (ng/mL)
Control females	5	3.38 ± 0.21^a^	205.04 ± 13.56^c^	603.56 ± 53.21^a^	2.74 ± 0.47
Ovarian hydrobursa	19	2.17 ± 0.07^cd^	425 ± 46.29^bc^	64.4 ± 24.38^b^	1.36 ± 0.28
Inactive ovaries	18	1.98 ± 0.12^cd^	325.12 ± 35.67^bc^	89.73 ± 21.89^b^	1.22 ± 0.29
Uterine inflammation	28	2.13 ± 0.08^cd^	393.92 ± 31.78^bc^	144.48 ± 38.57^b^	2.41 ± 0.38
Cervicitis	4	2.79 ± 0.19^b^	577.22 ± 161.8^ab^	215.06 ± 185.17^b^	0.94 ± 0.86
Vaginitis	7	2.5 ± 0.23^bc^	670.92 ± 105.27^a^	218.71 ± 91.19^b^	2.28 ± 1.0
Fallopian tube adhesions	4	2.01 ± 0.05^d^	394.02 ± 57.93^bc^	629.15 ± 145.22^a^	1.42 ± 0.77

^a,b,c,d^Means in the same column with different superscripts significantly differ at P *<* 0.05. FSH=Follicle-stimulating hormone, SEM=Standard error of the mean

Estradiol-17ß levels exhibited the highest (p < 0.05) values in fertile (603.56 pg/mL) and infertile females (629.15 pg/mL) suffering from SA. Although differences among other infertile groups were insignificant, the females who were diagnosed with ovarian hydrobursitis (BU) revealed the lowest level of estradiol-17ß (Table-1). Progesterone levels were not different (p > 0.05) among groups, but females suffering from cervical inflammation revealed the lowest progesterone concentration (0.94 ng/mL).

As shown in Table-2, in normal fertile females, an obvious relationship between ubiquitin and FSH is non-existent. However, there are positive correlation coefficients between ubiquitin and FSH among IO (r = 0.46; p < 0.05), EN (r = 0.41; p < 0.05), and vaginal inflammation (r = 0.76; p < 0.05). In normal fertile females, the relationship between ubiquitin and estradiol-17ß was statistically significant (r = 0.85; p = 0.07), whereas the opposite trend, although not significant, was found in most cases of infertility.

**Table-2:** Correlations between ubiquitin versus FSH, progesterone, and estradiol 17-ß within the control and subfertility status of the female dromedary camels.

Female group	No. of female	Ubiquitin		

		FSH	Estradiol	Progesterone
Control females	5	0.14	0.85 (p = 0.04)	−0.71
Ovarian hydrobursa	19	0.24	−0.22	−0.16
Inactive ovaries	18	0.46 (p = 0.043)	−0.03	−0.32
Uterine inflammation	28	0.42 (p = 0.028)	−0.04	0.27
Cervicitis	4	0.39	−0.81	−0.72
Vaginitis	7	0.76 (p = 0.046)	−0.3	−0.22
Fallopian tube adhesions	4	−0.35	−0.31	−0.91

FSH=Follicle-stimulating hormone

Table-3 illustrates the possible correlation coefficients between FSH and progesterone and estradiol-17ß. Although the values were not statistically significant, the relationship between FSH and estradiol-17ß in normal fertile females was positive; the relationship between FSH and progesterone was negative. The relationship between FSH and estradiol-17ß was found to be negative in females with ovarian BU, CE, vaginitis, and SA. Females with vaginal inflammation were the only subfertility category with a significant relationship (r = −0.67; p = 0.05). None of the animal categories have shown a significant relationship between FSH and progesterone.

**Table-3:** Correlations between FSH versus progesterone and estradiol 17-ß within the control and subfertility status of the female dromedary camels.

Female group	No. of female	FSH	

		Estradiol	Progesterone
Control females	5	0.62	−0.74
Ovarian hydrobursa	19	−0.29	−0.06
Inactive ovaries	18	0.02	0.05
Uterine inflammation	28	0.21	0.22
Cervicitis	4	−0.81	0.35
Vaginitis	7	−0.67 (p = 0.07)	−0.56
Fallopian tube adhesions	4	−0.31	0.2

FSH=Follicle-stimulating hormone

The relationship between progesterone and estradiol in normal fertile females was highly negative (r = −0.97; p < 0.01). In contrast, this relationship turned out to be highly positive in five out of the six cases of subfertility (Table-4).

**Table-4:** Correlations between progesterone and estradiol 17-ß within the control and subfertility status of the female dromedary camels.

Hormone	Progesterone						

	Control fertile females	Ovarian hydrobursa	Inactive ovaries	Uterine inflammation	Cervicitis	Vaginitis	Fallopian tube adhesions
Estradiol 17-ß	−0.97 (p = 0.005)	0.43 (p = 0.05)	0.47 (p = 0.038)	0.71 (p < 0.0001)	0.99 (p = 0.009)	0.88 (p = 0.009)	0.64

## Discussion

Several studies have been published elucidating the impact of heat stress on an animal’s productivity and reproductive performance [13–18]. The Arabian Peninsula’s harsh summer climates, with temperatures reaching above 50°C and drought conditions, imposed significant burdens on camel physiology and feed efficiency [19]. In addition, due to the incidence of malpractice among camel owners, especially those who are illiterate, pregnancy failures in female dromedaries occur. Historically, for example, the Bedouins who live in the desert and own major camel herds used to put pieces of hot peppers or even some ground black pepper in the female vagina after natural copulation, believing that this would accelerate the entrance of semen to achieve conception. Such malpractice negatively affects the health of the female genital tract, leading to irritations, inflammations, and adhesions in the vagina, cervix, and uterus. Frequent application of such irritants following natural mating invites bacterial infection that accumulates purulent fluids within the uterus, leading to uterine growth, which mimics pregnancy, leading the owners to believe that their females are pregnant. Ali *et al*. [20] investigated the causes of subfertility in 447 female camels that were categorized into three groups: Repeat breeders (76.7%), refused mating (16.3%), and early embryonic death (6.9%). They found that EN and metritis represented 57% of the tested cases that confirm the above malpractice by camel owners.

Furthermore, the lack of green forages in the desert and the animal dependence on rangeland herbs, which are nutrient deficient, in addition to providing the concentrated pellets or barley grains at low shares, cannot cover the nutritional requirements of the females. This also caused smooth inactive ovarian structures. The incidence of vaginal (VA) and cervical (CE) inflammations and adhesions were positively correlated with the high levels of ubiquitin in the blood circulation; furthermore, other subfertility categories demonstrated the role of inflammation on ubiquitin production [8]. In their recent study on 870 infertile female dromedaries, Ali and Derar [4] reported significant relationships between ovarian BU and overgrown ovarian follicles. Cytokines, including ubiquitin, were previously considered critical factors involved in several biological processes [21]. Ubiquitin was found to be a peptide responsible for several biological mechanisms, including cell signaling as an immune response [22].

Moreover, the ubiquitin-proteasome pathway is involved in protein degradation [23]; thus, it increases in cases of inflammation and immune diseases. This is demonstrated in the present study’s case, since the six categories of infertility in female camels expressed relatively higher ubiquitin concentrations when compared with CON females. The apparent reason for such increases in diseased females is to combat inflammation, adhesions, EN, and cystic ovarian structures [8]. In addition, the decrease in ubiquitin was paralleled by an increase in FSH in fertile females; however, the opposite was found in all diseased groups. The high concentrations of ubiquitin in diseased females could negatively influence the gonadotroph cells within the pituitary gland, leading to the observed reductions in FSH and estrogen secretion.

Nonetheless, in this respect, the hypothalamic-pituitary-ovarian imbalance cannot be overlooked. This finding was confirmed in humans by Amita *et al*. [24], who attributed the declines in estrogens to the role played by the ubiquitin-proteasome pathway. In the normal fertile camel females, the relationship between the two sex steroid hormones (i.e., estradiol and progesterone) was negatively correlated (r = −0.97). In all infertile categories, this relationship turned out to be positively correlated, which indicates imbalances in the function of the hypothalamic-hypophyseal-ovarian axis. Recently, it has been emphasized in humans that the overdominance of estradiol on progesterone leads to EN, inflammation, and endometrial receptivity to embryonic implantation [25]. The polycystic or smooth IO is a consequence of the endocrine imbalance, which is apparently happening in the current infertile cases as a response to the heat burden, lack of green forages, and less nutritional provision.

## Conclusion

Raising camels in the desert requires indoor housing in harsh climates, either cold or hot. Furthermore, the specific nutrient requirements of female camels at various physiological states should be highlighted. Further studies are required to determine the association of the oxidant parameters with the clinical subfertility status and reproductive hormones. In addition, training workshops are being conducted for camel owners in the Gulf region, guiding them toward the proper handling, practices, and sanitary measures to keep their animals healthy and productive.

## Authors’ Contributions

YMA: Sole author of this study, as he conceptualizes the idea, carried out the field and laboratory work, analyzed the data, and wrote the manuscript. The author has read and approved the final manuscript.
